# Effects of AM80 compared to AC261066 in a high fat diet mouse model of liver disease

**DOI:** 10.1371/journal.pone.0211071

**Published:** 2019-01-24

**Authors:** Marta Melis, Xiao-Han Tang, Steven E. Trasino, Viral M. Patel, Daniel J. Stummer, Jose Jessurun, Lorraine J. Gudas

**Affiliations:** 1 Department of Pharmacology, Weill Cornell Medicine, New York, NY, United States of America; 2 School of Urban Public Health, Hunter College, City University of New York, New York, NY, United States of America; 3 Department of Pathology, Weill Cornell Medicine, New York, NY, United States of America; 4 Weill Cornell Graduate School of Biomedical Sciences, New York, NY, United States of America; Laboratoire de Biologie du Développement de Villefranche-sur-Mer, FRANCE

## Abstract

The roles of retinoids in nonalcoholic fatty liver disease (NAFLD) remain unclear and a better understanding may lead to therapies that prevent or limit NAFLD progression. We examined the actions of retinoic acid receptor (RAR) agonists- AM80 for RARα and AC261066 for RARβ2- in a murine model of NAFLD. We fed wild type C57Bl/6 mice a chow or a 45% high fat diet (HFD) for 12 weeks, followed by 4 additional weeks with the HFD+AM80; HFD+AC261066; or HFD. The HFD+AM80 group showed greater hyperglycemia and glucose intolerance compared to other groups. Histopathological evaluation of the livers showed the highest degree of steatosis, triglycerides levels, and inflammation, assessed by F4/80 staining, in the HFD+AM80-treated compared to the HFD, the HFD+AC261066, and chow-fed mice. Liver vitamin A (retinol (ROL)) and retinyl palmitate levels were markedly lower in all HFD groups compared to chow-fed controls. HFD+AC261066-treated mice showed higher levels of a key intracellular ROL transporter, retinol-binding protein-1 (RBP1) compared to the HFD and HFD+AM80 groups. In conclusion, these data demonstrate that the selective RARα agonist AM80 exacerbates HFD-induced NAFLD and hyperglycemia. These findings should inform future studies examining the therapeutic potential of RAR agonists in HFD-related disorders.

## Introduction

Vitamin A (VA, all-trans retinol) is an essential nutrient involved in regulation of numerous biological pathways during development and adult life [1–6]. VA is metabolized to all-trans retinaldehyde, and then to all-trans retinoic acid (RA) in target cells [7, 8], where, through its cognate RA nuclear receptors α, β, and γ (RARα (NR1B1); RARβ (NR1B2); and RARγ (NR1B3)), RA regulates the transcription of cell and context-specific genes and gene pathways [9]. Each of the RAR genes generates a number of protein isoforms, (e.g. RARβ1, RARβ2, RARβ3), from transcripts initiated at two different promoters [10]. The RARs α, β, and γ exhibit different tissue and cell-type functions [1], and regulate the transcription of different genes in various cell types, even though all three receptors bind the endogenous agonist RA [10–16]. RA and RARs regulate a number of pathways implicated in obesity-related disorders such as type 2 diabetes (T2D) and non-alcoholic fatty liver disease (NAFLD) [4, 16, 17]. Our research, coupled with a growing body of data, demonstrate that obesity and its related disorders, such as NAFLD, nonalcoholic steatohepatitis (NASH), and T2D, are associated with reduced tissue levels of vitamin A (retinol, ROL) and retinyl esters in mice [18] and in humans [19]. Given the reduced VA in obesity-related diseases, a potentially novel therapeutic approach is the use of highly selective agonists for RARα, β, and γ.

We recently demonstrated that administration of a highly selective RARβ2 agonist, AC261066 [20], reduced hyperglycemia and hepatic steatosis in several mouse models of type 2 diabetes (T2D) and NAFLD [16, 17]. Here we compared this selective agonist for RARβ2, AC261066, to a selective agonist for RARα, AM80 [21–23], in a high fat diet (HFD)-induced murine model of NAFLD. We found that AC261066 and AM80 exhibited opposing effects, and that AM80 enhanced HFD-induced NAFLD.

## Materials and methods

### Methods

The experiments were conducted in accordance with the Institutional Animal Care and Use Committee (IACUC) guidelines at Weill Cornell Medical College (WCMC) in accordance with all applicable federal, state and local regulations. All experimental protocols for this specific study were approved by IACUC (animal use protocol number 0705-615A) including animal care, housing, and sanitization (WCMC Animal Welfare Assurance Number: D16-00186); WCMC Institutional Biosafety Committee (IBC) Laboratory Registration #: IBC-18783.

### Animals and treatments

Wild-type (wt) C57BL/6 male mice (Jackson Laboratory, Bar Harbor, ME) were kept on 12-hour light/dark cycles. At 6 weeks of age they were either fed ad libitum a chow diet with 13% of the kcal from fat (cat# 5053, 15 IU of vitamin A-acetate/gram, Test-Diet, Inc.), or a high fat diet (HFD), with 45% of the kcal from fat (cat# 58125, 4.7 IU vitamin A-acetate/gram, Test-Diet, Inc.). We chose these chow and high fat content diets based on evidence previously published by our group [18] indicating that wild-type C57BL/6 mice fed a high fat diet (HFD) with 4.7 IU vitamin A-ester/gram (#58125,Test-Diets Co, St. Louis, MO) showed equal reductions in tissue vitamin A levels compared to control mice fed either a custom control diet (#58124, Test-Diets Co, St. Louis, MO), that contains 3.8 IU vitamin A-ester/gram), or the WCMC vivarium rodent diet with 15 IU vitamin A-ester/gram (Pico Rodent Diet, #5053) [18]. In the present study, the mice fed a HFD for 3 months were divided into three groups, each treated for an additional month on the HFD with (a) no additional drugs (vehicle, 0.6% DMSO); (b) the RARα synthetic agonist, 4-[(5,6,7,8-Tetrahydro-5,5,8,8-tetramethyl-2-naphthalenyl)carboxamido]benzoic acid (AM80) (cat# S4260, lot n.01, Selleck Chem), at a concentration of 0.4 mg/100 mL (~0.6–0.8 mg/Kg bw) in 0.6% DMSO, or (c) the RARβ2 synthetic agonist, 4-(4-(2-butoxyethoxy)-5-methylthiazol-2-yl)-2-fluorobenzoic acid (AC261066) (cat# 4046, R&D Systems) [20], at the concentration of 3 mg/100 mL (5.3 mg/Kg bw) in 0.6% DMSO, with both drugs administered in the drinking water (n = 4 mice per group).

### Glucose tolerance tests (GTT)

After four months of treatment, and after fasting overnight, we tested the cohorts of mice to measure their clearance rate of blood glucose. Briefly, we challenged the mice with an injection of a 20% glucose solution in PBS at 2.0 g/kg of body weight (n = 4 mice per group). We measured blood glucose from tail veins at 15, 30, 45, 60, and 120 minutes post injection using a Free Style Lite Blood Glucose Monitoring System (Abbott Diabetes Care, Inc.).

### High performance liquid chromatography (HPLC)

We sacrificed the mice by cervical dislocation, strictly following the guidelines approved by the IACUC. We collected mouse blood and we used 50 μl of serum for retinoid extraction. We homogenized mouse tissues (50 mg) in ice-cold PBS. For retinoid extraction, we added 350 μl of acetonitrile-butanol (50:50, v/v) and 0.1% butylated hydroxytoluene to a total volume of 0.5 ml of tissue homogenate in PBS. We vortexed the mixtures thoroughly for 60 seconds and added one external standard, retinyl acetate, to homogenates to assess recovery levels (mice do not synthesize retinyl acetate, so retinyl acetate is not one of the endogenous retinoids). After the addition of 300 μl of a saturated (1.3 kg/l) K_2_HPO_4_ solution and thorough mixing, we centrifuged the samples for 10 min at 12,000 G at room temperature. We next collected the upper organic layers and transferred them for HPLC analysis. To separate the various retinoids, we performed HPLC analysis using a Waters Millennium system (Waters Corp., Milford, MA). Samples were applied to an analytical 5 μm reverse-phase C18 column (Vydac, Hesperia, CA) at a flow rate of 1.5 ml/min. We identified the different retinoids by HPLC based on two criteria: an exact match of the retention times of unknown peaks with those of authentic retinoid standards, and identical ultraviolet light spectra (220–400 nm) of unknowns against spectra from authentic retinoid standards during HPLC as measured by a photodiode array detector. Finally, we normalized the absorbance values to the tissue weights.

### Histology and pathologic examination

We fixed liver and pancreas samples in 4% formaldehyde buffer and embedded them in paraffin blocks. Next, we stained 5 μm sections with hematoxylin and eosin (H&E) and a liver pathologist (JJ) evaluated each sample in a blinded manner for evidence of steatosis, fibrosis, and steatohepatitis, based on best clinical practice and the Brunt criteria [24]. The main histopathological features considered for each section included: ductular reaction (score 0–3), portal and lobular inflammation (0- none, 1-mild, 2-moderate, 3-severe), presence or absence of atypical cells comprising the ductular reaction, degree of steatosis (0-none, 1<30%, 2>30 but <60%, 3- >60%), type of steatotic vacuoles (microvesicular or macro vesicular) and ballooning degeneration (0-none, 1-occasional, 2-more than occasional, 3-numerous cells).

### Serum triglycerides measurements

To measure fasting serum triglycerides, we used a CardioChek PA hand held lipid analyzer and PTS triglyceride test strips (Polymer Technology Systems, Inc., Indianapolis, IN). We performed this test in 4 mice per group.

### Liver triglyceride extraction

To quantify the hepatic levels of triglycerides, we used the Folch method [25] with 4 mice per group. We extracted the liver lipids (50 mg tissue) with a mixture of chloroform and methanol (2:1 ratio). To dry the lipid extracts we used nitrogen gas, followed by resuspension in 200 μl isopropanol and quantification using the Liver Triglycerides kit (ThermoFisher Infinity Kit, cat# TR22421) on a SpectraMax M2e microplate reader (Molecular Devices, Inc.) according to the manufacturers’ protocol.

### Immunofluorescence microscopy

We fixed liver tissues overnight with 4% formaldehyde at 4°C, followed by treatment with 25% sucrose and optimal cutting temperature (OCT) embedding, before cryostat-sectioning. Next, we blocked 3–4 slides per group with 2% bovine serum albumin (BSA) in PBS/Triton X 0.1% or a cocktail of mouse IgG (cat# MKB-2213, lot# ZB0618, diluted 1:30, Vector Labs, Inc.) and blocking serum, if using mouse antibodies, for 1 hour at room temperature (rt). After optimization of the staining protocol, we incubated the slides for 4 hours at 37°C with mouse anti-RBP1 (CRBP1) (cat# sc-271208, lot# B0912, 1:50) (Santa Cruz, Inc.) or overnight at 4°C with rat-anti F4/80 (Bio Legends, cat. n. 123101, Lot. B226028, 1:100). To assess non-specific staining, we included a negative control slide incubated without primary antibody. After rinsing in PBS, we then incubated the slides with Alexa Fluor 594 conjugated anti-mouse or Alexa Fluor 488 conjugated anti-rat (Invitrogen) secondary antibodies, diluted 1:500, for 1 hour at 22°C. We stained the nuclei with Hoechst Dye 33258 (cat# 382061, Calbiochem) at a concentration of 0.5 μg/mL in PBS for 1 minute, followed by one wash in PBS and mounting for image acquisition.

We acquired a minimum of 4 random fields per tissue section and quantified the level of fluorescence with Fiji (Image J) software (v1.48, NIH) according to previously published criteria [26]. Briefly, we calculated the relative level of fluorescence as integrated density–(area of selected cell × mean fluorescence of background readings). The data were then represented as mean ± standard deviation (SD).

### RNA isolation and quantitative RT PCR (qRT-PCR)

We extracted total RNA with TRI Reagent (Sigma Aldrich) from mouse liver (3–4 mice per experimental group) and used 1 μg RNA to synthesize complementary DNA (cDNA) with random primers. To assess the level of gene expression we used specific primers for Retinol Binding Protein 1 (RBP1) (Gene ID: 19659) Forward primer (5’– 3’): GCTGAGCACTTTTCGGAACT; Reverse primer (5’– 3’): GGAGTTTGTCACCATCCCAG. The housekeeping gene used was the ribosomal phosphoprotein P0 (RPLP0) indicated as 36B4 (Gene ID: 11837) Forward primer (5’– 3’): AGAACAACCCAGCTCTGGAGAAA; Reverse primer (5’– 3’): ACACCCTCCAGAAAGCGAGAGT. We performed qRT-PCR as previously described [27] using SYBR Green PCR master mix on a Bio-Rad MyiQ2 Real Time PCR iCycler (Bio-Rad). To calculate the different gene expression in the experimental groups we used the delta Ct method and normalized to the expression of the internal control gene 36B4 [17].

### Statistics

The data are represented as the mean ± standard deviation (SD). We determined the statistical significance between groups by one-way ANOVA and Bonferroni multiple comparison post-hoc analysis (black stars), *P* values of < 0.05 were considered statistically significant. We performed all statistical analyses using GraphPad Prism 6.0 software (GraphPad Software, San Diego, CA).

## Results

### AM80 and AC261066 exert different effects on glucose levels in HFD-fed mice

**Fig 1:**
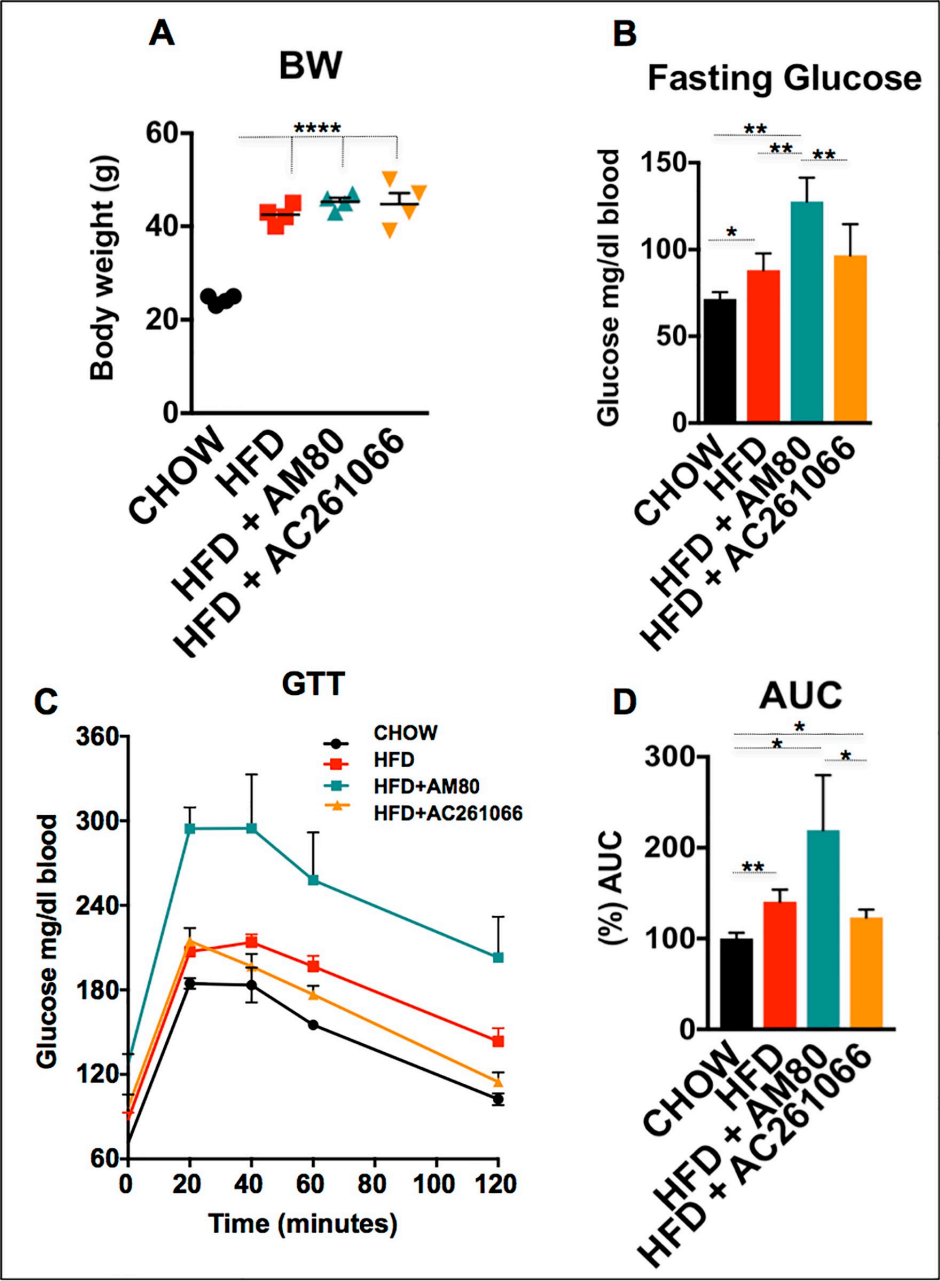
The RARα agonist AM80 does not influence the body weight of HFD-fed mice, but increases the levels of fasting glucose and glucose intolerance. (A) The body weights (bw) of mice that received HFD alone, HFD+AM80, or HFD+AC261066 were increased as compared to the chow-fed mice. (B) Levels of fasting glucose in the HFD-, HFD+AM80-, and HFD+AC261066-treated as compared to chow-fed mice. (C) Glucose tolerance tests (GTT), performed after overnight fasting, with an intraperitoneal (ip) injection of 20% glucose in PBS, at 2 g glucose/Kg. (D) The quantification of the GTT is represented by the area under the curve (AUC) plot. Mice used in all experiments = 4 per group. The plots are represented as mean ± standard deviation (SD). Black stars represent statistical significance according to the one-way ANOVA test, p < 0.05 = *; p < 0.01 = **; p < 0.001 = ***; p < 0.0001 = ****.

We previously showed that administration of synthetic agonists of RARβ2 to wild type C57Bl/6 mice in a high fat diet (HFD) model of nonalcoholic fatty liver disease (NAFLD) reduced liver steatosis, inflammation, and oxidative stress [16, 17]. These results prompted us to investigate whether or not other selective, synthetic agonists of other RARs can influence the development of NAFLD. Therefore, we fed 6-week-old C57Bl/6 male wild type (wt) mice a chow diet (13% kcal/fat) or a HFD (45% kcal/fat) for 12 weeks, followed by 4 weeks during which we continued to feed the mice a HFD, but also added either an RARα agonist, AM80 [28], or the RARβ2 agonist AC261066 [16, 20], in the drinking water. We observed increased body weights (bw) in the mice fed a HFD, a HFD+AC261066, and a HFD+AM80 compared to mice fed a chow diet (p < 0.0001) (**Fig 1A**). We also assessed the levels of serum triglycerides in all experimental groups. We observed no statistically significant differences across the experimental groups (**S1 Fig**), indicating no impact of AM80 or AC261066 on the levels of serum triglycerides after a 4-week treatment.

Next, we tested whether AM80 and AC261066 affected the blood glucose levels in the mice, as this represents one metabolic parameter that is consistently altered in mouse models of NAFLD [6, 16, 29] and in a large number of patients with NAFLD [30–33]. Genetically manipulated obese mice, such as the *ob/ob* and *db/db* strains, and wild type mice fed a HFD progressively develop severe hyperglycemia and impaired glucose responsiveness, two clinical hallmarks in the development of type 2 diabetes (T2D) [16, 34].

We performed glucose tolerance tests (GTT) on the cohorts of mice that had been fasted overnight. The HFD+AM80 treated group showed the highest fasting glucose levels, which corresponded to time zero in this test (**Fig 1B**), and exhibited twice the blood glucose levels (p < 0.001) of the chow-fed mice. We also found that the HFD+AM80 treated mice exhibited a greater delay in glucose clearance (**Fig 1C**), and area under the curve (AUC) glucose levels (**Fig 1D**) compared to both the HFD and HFD+AC261066 groups. These results indicate that treatment of HFD-fed mice with a selective RARα agonist, AM80, results in more pronounced hyperglycemia and glucose intolerance as compared to the chow, HFD, and the HFD+AC261066 treated mice.

### Retinol levels are lower in livers of HFD, HFD+AM80, and HFD+AC261066 treated mice

**Fig 2:**
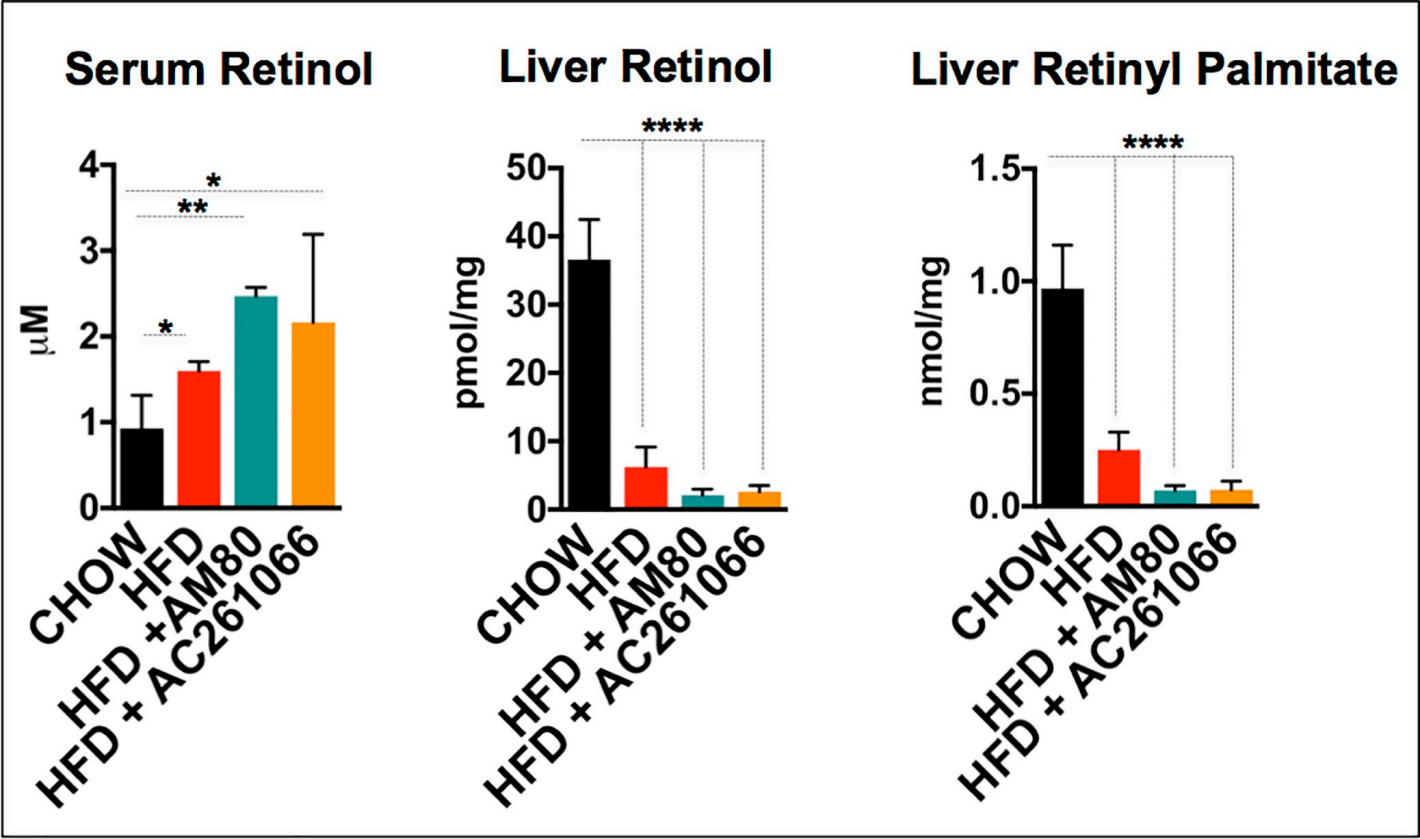
Retinol (ROL) is increased in serum, and retinol and retinyl palmitate are decreased in HFD-, HFD+AM80- and HFD+AC261066-treated compared to chow-fed mice. Quantitation by HPLC of serum retinol, liver retinol, and liver retinyl palmitate in chow-, HFD-, HFD+AM80-, and HFD+AC261066-treated mice. The plots are represented as mean ± standard deviation (SD). Mice per group = 4; Black stars represent statistical significance according to the one-way ANOVA test, p < 0.05 = *; p < 0.01 = **; p < 0.001 = ***; p < 0.0001 = ****.

We measured the ROL levels in the sera and livers, and the retinyl palmitate (RP) levels in the livers of these mice by HPLC analysis. We found that the serum ROL levels in the HFD, HFD+AC261066, and HFD+AM80 treated mice were increased relative to the chow-fed mice (**Fig 2**). In sharp contrast to the serum ROL data, the HFD, HFD+AM80, and HFD+AC261066 fed mice showed major decreases in liver ROL and RP levels compared to the levels in the livers of the chow-fed mice **(Fig 2)**. These data are consistent with our previous, published data showing that obesity dramatically lowers retinol levels in tissues, but not in serum, even though these high fat diets contain adequate amounts of retinol as retinyl acetate or retinyl palmitate [18].

**Fig 3:**
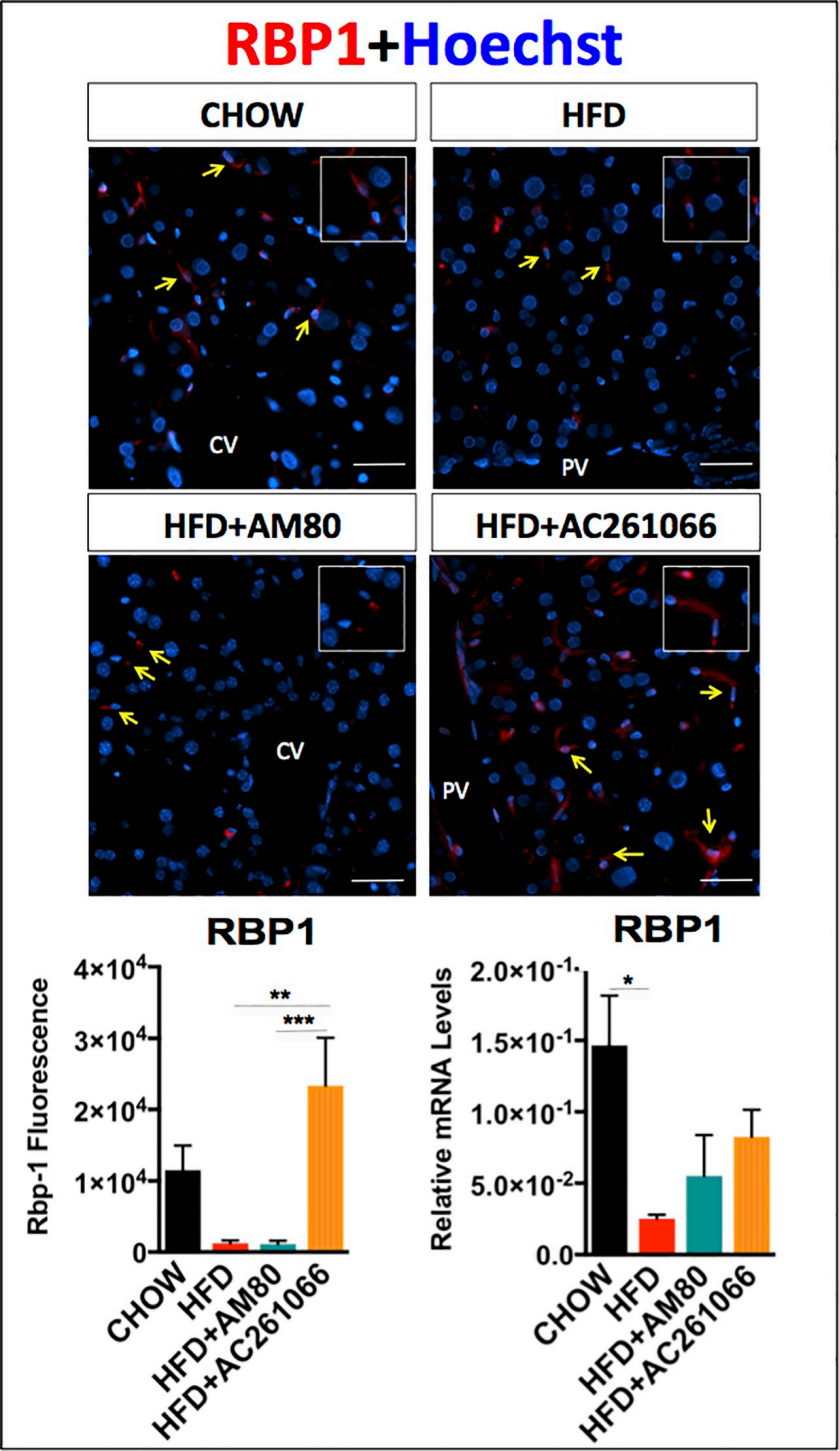
The RARβ2 agonist AC261066 increases the hepatic levels of RBP1 in HFD-fed mice. Representative images depicting RBP1 in red and nuclei in blue (Hoechst labeling) assessed by immunofluorescence in frozen liver tissues and by qRT-PCR in chow-, HFD-, HFD+AM80-, and HFD+AC261066-treated mice. Yellow arrows in the images indicate representative areas of RBP1-positivity (inserts). Quantification of the immunofluorescence and mRNA levels is represented as a bar plot. The plots are represented as mean ± standard deviation (SD) (mice per group = 3–4; image fields per mouse > 4). Statistical significance is assessed by one-way ANOVA (black stars), p < 0.05 = *; p < 0.01 = **; p < 0.001 = ***. Magnification = 200X. Scale bar = 100 μm. CV = central vein; PV = portal vein.

To characterize further the effects of AM80 and AC261066 in this NAFLD model we measured the hepatic expression of retinol binding protein-1, RBP1, a key marker of hepatic stellate cells (HSCs), which represent the main vitamin A storage compartment in liver [35]. RBP1 is involved in the intracellular transport of vitamin A (retinol, ROL) [36, 37]. Despite being the main vitamin A storage compartment, HSCs make up only 10% of the liver resident cells and 6% of the organ volume [38]. Therefore, detection of HSCs by immunostaining allowed us to examine their distribution throughout the liver parenchyma. Morphological evaluation of the RBP1 staining showed most of the positivity in the sinusoidal space adjacent to the hepatocytes (Fig 3), where the HSCs normally reside [39], which is consistent with our previous work [18] and other research [40, 41]. We also observed that sinusoidal regional RBP1 expression distribution was relatively uniform across periportal, pericentral and centrilobular sinusoid regions among all groups (Fig 3). We previously reported that RBP1 is markedly decreased in both mice and human livers with increasing severity of NAFLD [18]. By immunofluorescence we found that hepatic RBP1 protein levels in the HFD-fed and the HFD+AM80 treated mice were 8.6-fold (±1.1) (p > 0.05) and 9.3-fold (±1.4) (p > 0.05) lower, respectively, than those in the chow-fed mice (**Fig 3**), whereas the levels of RBP1 protein in HFD+AC261066 treated mice were >18-fold (± 1) (p < 0.01) higher than those in the HFD and the HFD+AM80 treated mice (**Fig 3**). Mice on a HFD also showed a 6-fold (± 0.17) (p < 0.05) lower RBP1 mRNA level than that observed in chow-fed mice. However, treatment of the mice on the HFD with AC261066 resulted in a 2-fold (± 0.4) increase in RBP1 mRNA compared to the HFD treated group **(Fig 3)** (not significant, p > 0.05). In contrast, we observed no changes in the RBP1 mRNA levels in the HFD+AM80 treated mice compared to those in the HFD treated mice **(Fig 3).**

### Effects of AM80 and AC261066 on hepatic lipid levels

**Fig 4:**
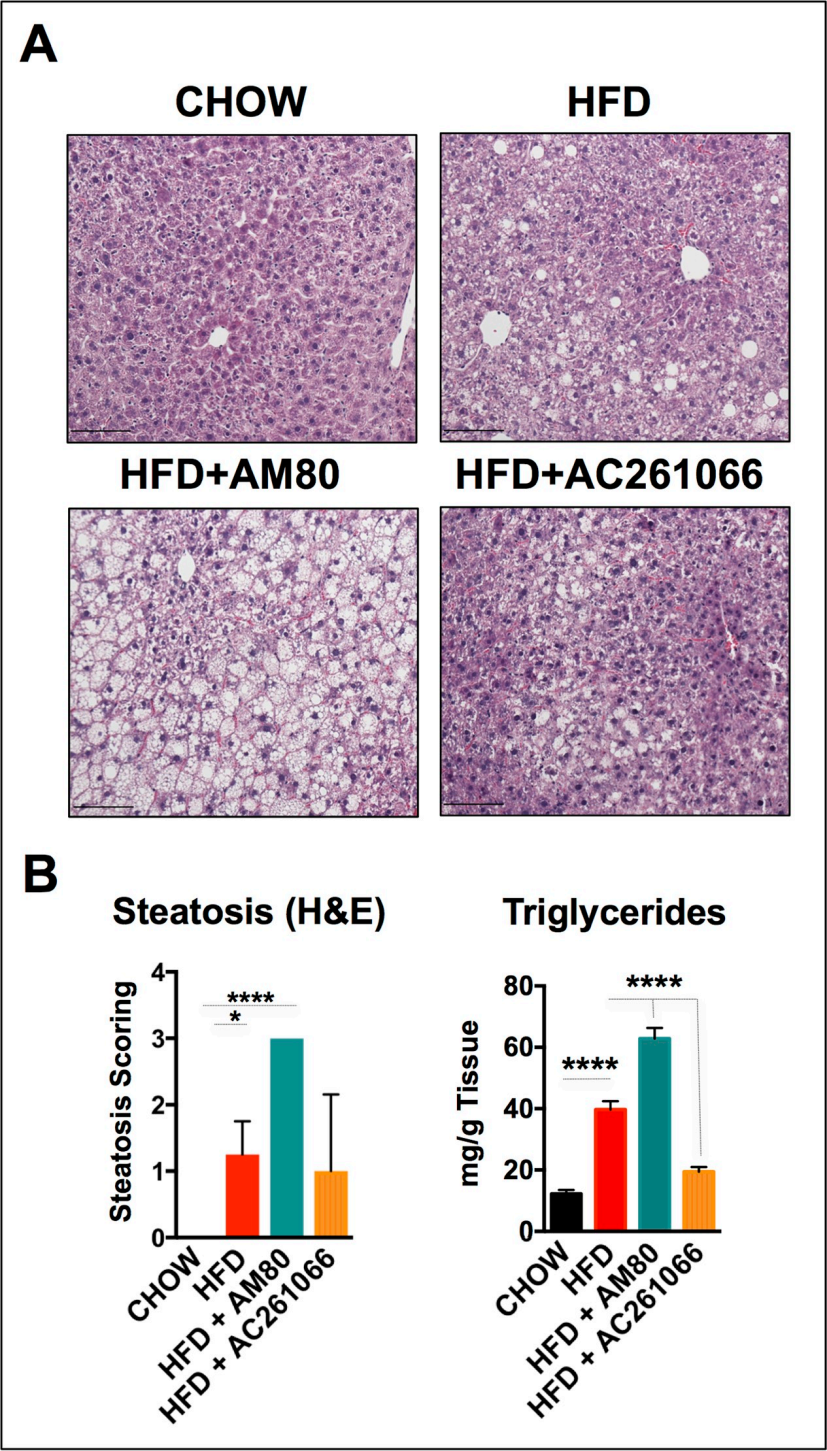
The RARα-synthetic agonist AM80 increases steatosis in HFD-fed mice. **(A)** Representative images of hematoxilin and eosin (H&E)-stained liver sections in chow-fed and HFD-, HFD+AM80-, and HFD+AC261066-treated mice. (**B**) Scoring of steatosis was assessed according to the Brunt criteria (details in the methods) in the H&E images (4 mice per group) and lipid accumulation by Folch extraction (triglycerides) (4 mice per group). The plots are represented as mean ± standard deviation (SD). Statistical significance is assessed by one-way ANOVA (black stars), p ≤ 0.05 = *; p ≤ 0.0001 = ****. Magnification 200X. Scale bar = 100 μm.

Because hepatic steatosis is one of the key phenotypic indicators of NAFLD, we next performed histopathological evaluation of steatosis according to the Brunt criteria [24]. The steatosis level (range 0 to 3) in all of the chow-fed mice was scored as 0. The livers of the HFD-fed mice ranged from 1 to 2, with all of the four mice analyzed showing macro-vesicular steatosis (**Fig 4A**), whereas livers of the HFD+AC261066 treated mice showed a heterogeneous pattern ranging from no fat deposition, 0, to moderate, 2, with some micro- and macro-vesicular steatosis in 2 of the 4 mice analyzed (**Fig 4A**). The livers of the HFD+AM80 treated mice showed the highest levels of steatosis; all livers scored a maximum steatosis level, 3, with micro-vesicular steatosis in 3 of the 4 mice analyzed (**Fig 4B**).

We quantified the liver triglyceride content, as described in the Methods section [25], and found a 2.8-fold (± 0.14) (p < 0.0001) increase in triglyceride levels in the HFD compared to the chow-fed mice (**Fig 4B**). In line with our previous steatosis assessments, the HFD+AM80 group showed the highest triglyceride levels compared to the chow-fed mice, a 4-fold (± 0.11) (p < 0.0001) increase, whereas the HFD+AC261066 treated mice showed an increase of less than 1-fold (± 0.17) (p < 0.01) compared to the chow-fed mice (**Fig 4B**). Taken together, these data show that the HFD+AM80 group exhibited a more severe NAFLD phenotype in terms of hepatic lipid content than the HFD and the HFD+AC261066 groups.

### Effects of AM80 and AC261066 on inflammation

**Fig 5:**
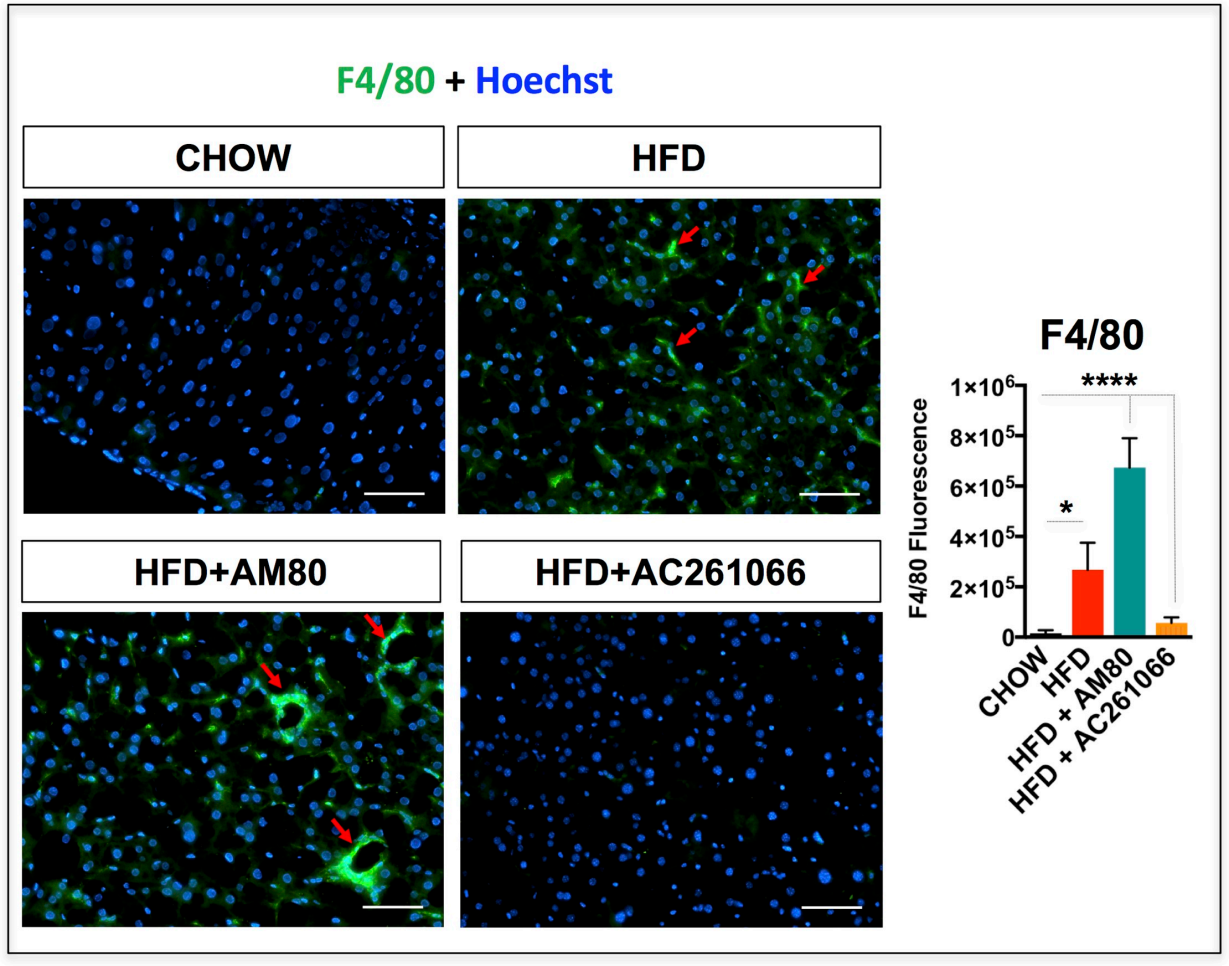
The RARα-synthetic agonist AM80 increases levels of F4/80 in HFD-fed mice. Representative images depicting hepatic Kupffer cells stained with the F4/80 marker in green and nuclei in blue (Hoechst labeling) in chow-, HFD-, HFD+AM80-, and HFD+AC261066-treated mice assessed by immunofluorescence in frozen liver tissues. Red arrows indicate representative areas positive for F4/80. The plots are represented as mean ± standard deviation (SD); Mice per group = 4; Image fields measured per mouse > 4; Statistical significance is assessed by one way ANOVA (black stars), p < 0.05 = *; p < 0.01 = **; p < 0.0001 = ****. Magnification = 200X. Scale bar = 100 μm.

NAFLD is a hepatic manifestation of the metabolic syndrome, which involves multiple organs and presents a broad spectrum of histologic and functional changes in the liver [42]. While NAFLD in humans can be a benign and asymptomatic condition for decades, it is often associated with chronic, low-grade hepatic inflammation [43], which over time can contribute to the development of a more severe form of NAFLD and NASH [44]. The pan-marker of murine macrophages, F4/80 [45], is a reliable indicator of hepatic inflammation and is detected starting at 6 weeks to 16 weeks of HFD treatment in C57Bl/6 mice [46]. We previously reported that the RARβ2 agonist AC261066, administered for 3 months to mice on a HFD, attenuated hepatic expression of F4/80 compared to untreated HFD-fed mice [17]. Here we compared the effects of a one-month treatment with AC261066 and AM80 on hepatic expression of the macrophage marker F4/80 in this NAFLD model. By immunofluorescence, we found that the HFD-fed mice showed a 16-fold (± 1.7) (p < 0.05) increase in hepatic F4/80 protein compared to chow-fed mice (**Fig 5**). The HFD+AM80 treated and the HFD+AC261066 treated mice showed 42-fold (± 0.39) (p < 0.0001) and 2.7-fold (± 1.22) (not significant, p > 0.05) increases, respectively, compared to the chow-fed mice (**Fig 5**).

These data indicate that a one-month treatment with the RARβ2 agonist, AC261066, exerted anti-inflammatory effects on mice fed a HFD. In contrast, treatment with the RARα agonist, AM80, for one month in mice fed a HFD increased the hepatic levels of inflammation, underscoring key differences in the metabolic modulating properties of these two synthetic retinoid agonists in the liver.

## Discussion

NAFLD is generally the hepatic manifestation of a much broader spectrum of disorders, i.e. hyperglycemia, type 2 diabetes, and cardiovascular disease, all of which contribute to the metabolic syndrome [47]. There is a growing body of data demonstrating the potential of natural and synthetic retinoids as a novel class of drugs for management of metabolic disorders because of their ability to favorably regulate liver energy metabolism and energy expenditure [48, 49]. In support of this, studies of mice treated with high doses of RA, 10, 50, and 100 mg/kg bw, for 4 days show increased fatty-acid oxidation in skeletal muscle [49], reductions in body weight, and decreased adiposity compared to untreated mice [50]. In addition to the actions of RA in extrahepatic tissues, in diet-induced obesity models mice treated with a high dose, 15 mg/kg, of RA by gavage for 7 days had decreased hepatic steatosis compared to untreated mice [51].

We have recently shown both in diet-induced and genetic obesity models that agonists for RARβ2, but not RARγ, decreased hepatic steatosis, inflammation, and hyperglycemia [16]. The two most abundant RARs in liver are RARα and RARβ [52, 53]. Therefore, selective activation of one of the RARs, namely RARβ2, may represent a more targeted approach to prevent or reverse NAFLD. This prompted us to investigate whether activation of RARα can influence the development of NAFLD. In the research presented here, we dissected the roles of two RAR agonists with high affinity for RARα (AM80) and RARβ2 (AC261066) in NAFLD. By HPLC we assessed the AM80 concentration in the HFD+AM80-treated mice that received the drug for one month in the drinking water and detected AM80 at a concentration of 1.1 nM in the liver of one mouse, whereas in the remaining three mice analyzed, the AM80 levels were below the detection threshold. Similarly, we assessed the AC261066 concentration in the HFD+AC261066-treated mice and detected AC261066 at a concentration of 5.4 nM in one mouse, whereas the AC261066 levels in the remaining three mice were below the detection threshold. These preliminary assessments provided evidence that AC261066 and AM80, supplied in drinking water, reached the livers. Importantly, based on previous research [20], the EC50 of AC261066 for RARβ2 is 7.9 nM, which makes AC261066 selective for RARβ2 over RARα (EC50 = 631 nM) and RAR γ (EC50 = 501 nM). Moreover, Tacke et al. [28] showed that 1 nM AM80 was not able to activate RARβ. Taken together, these data indicate that the AM80 and AC261066 concentrations used in the present work should primarily activate the receptors RARα and RARβ2, respectively.

### AC261066 treatment increases hepatic expression of RBP1

Before being metabolized to RA, vitamin A (ROL) binds RBP1 [37, 54]. Our group previously demonstrated that obesity leads to decreased ROL and RP in mouse liver, pancreas, kidney, and lung, but not in serum [18]; moreover, marked reductions in hepatic RBP1 protein levels are seen in both murine and human NALFD [18]. In agreement with these data, we found higher serum ROL and lower ROL, RP, and RBP1 levels in the livers of HFD-fed mice compared to control chow-fed mice (**Fig 2**), reinforcing the concept that tissue vitamin A metabolism is altered in obesity-related disorders. However, despite the reductions in hepatic ROL and RP in HFD and in both RAR agonist treated HFD groups (**Fig 2**), we found that HFD+AC261066 treated mice, but not HFD+AM80 treated mice, exhibited markedly higher hepatic RBP1 protein levels relative to those in HFD-fed mice (**Fig 3**). Reductions in hepatic RBP1 in NAFLD have been demonstrated in our previous research and by others [18, 55]. One study reported increased hepatic RBP1 in human NAFLD [56], but as the authors’ highlight, increased hepatic RBP1 and other mediators of retinoid metabolism likely reflect increased flux of retinyl esters towards RA that has been observed in a number of liver diseases [56].

It is unclear if RBP1 itself possesses anti-NAFLD properties. RBP1 contains a retinoic acid responsive element (RARE) in its promoter [57]. Furthermore, RBP1 knockout mice demonstrate that RBP1 has an essential role in maintaining normal hepatic retinol storage pools and that RBP1 functions in part to slow hepatic retinol (ROL) turnover [36, 58]. Thus, our findings that AC261066, but not AM80, can uniquely improve NAFLD and increase hepatic RBP1 suggest that some of the anti-NAFLD properties of AC261066 may occur through canonical retinoid signaling and by reducing turnover and promoting the stabilization of existing hepatic ROL:RBP1 pools. These data may have important clinical relevance for the development of RARB2 agonists for treatment of NAFLD and NASH, as studies demonstrate that exogenous ROL and normalization of hepatic retinoid pools promote reversion of hepatic fibrosis [59]; furthermore, impaired hepatic RE hydrolase activity and mobilization of RE are associated with increased risk of NAFLD, NASH, and hepatocellular carcinoma (HCC) in mouse and human studies [19, 60].

We found that the mRNA RBP1 changes across groups mimicked the RBP1 changes occurring at protein level (**Fig 3**). Based on these evidence, We conclude that the relative differences in total hepatic RBP1 mRNA levels that we detected by qPCR (**Fig 3**) are primarily due to changes to HSC RBP1 expression for the following reasons: i) In all the RBP1-positive fields analyzed, across each experimental group, RBP1-positive cells were not detected outside of the liver sinusoids, anatomically excluding the involvement of hepatocytes; ii) The morphology of the RBP1 positive cells in our immunostaining is consistent with classic HSC morphology, based on their distinct spindle-to round shaped cell bodies with long branching membrane processes, which are not observed in hepatocytes. Taken together, our immunostaining results suggest that hepatocyte expression of RBP1 was below the level of detection, and thus, we concluded that hepatocytes made a negligible contribution to the relative changes to hepatic CRBP1 expression across experiment groups. This conclusion is consistent with the body of data across retinoid and pathology studies demonstrating that HSCs are the major CRBP1 expressing cells in rodent and human liver [35, 61, 62]. Nevertheless, we propose that future studies should consider hepatic isolation and analysis of purified pools of all major hepatic cell types (i.e. hepatocytes, HSCs, endothelial and Kupffer cells) in order to further the understanding of the hepatic cell type contributions to CRBP1 expression patterns in RAR-isotype agonist treated mice”.

### AM80 is associated with greater steatosis, hyperglycemia and glucose intolerance in a dietary murine obesity model

AM80 has been investigated in Acute Promyelocytic Leukemia (APL) [23, 63, 64], breast cancer [65], Type 1 Diabetes [21], Alzheimer’s disease [22], and autoimmune diseases [66–68], among other disorders. However, there are limited data on the role of AM80 in NAFLD [69]. In patients treated for APL, AM80 given at a dose of 0.08 mg/Kg for 41 to 58 days led to complete remission in a proportion of patients that relapsed after complete remission upon all-trans-Retinoic Acid (ATRA) treatment [23, 63, 64]. However, among the most common reported side effects were hypertriglyceridemia and hypercholesterolemia [23, 63, 64]. Our assessments of the levels of serum triglycerides in mice did not show any changes across experimental groups (**S1 Fig**). However, one of the key findings in our work is the higher level of liver triglycerides and steatosis in the HFD+AM80- compared to the HFD+AC261066- and HFD-treated mice (**Fig 4**). There are several possible explanations for why we failed to detect elevated levels of serum triglycerides in mice apart from species differences. For instance, we performed 4-week long treatments (30 days), versus up to 58 days of treatment for APL patients [63]. In addition to this, there is no information about the metabolic and liver status of patients that developed hypertriglyceridemia and hypercholesterolemia prior to starting the AM80 treatment.

### AM80 is associated with greater liver inflammation

Our data point to increased hepatic macrophage expression in the HFD-treated compared to chow-fed mice, as indicated by the positive F4/80 staining (**Fig 5**), indicative of inflammation, a hallmark of NAFLD [46]. However, in the HFD+AM80 group we observed greater macrophage F4/80 staining compared to HFD-treated mice, indicating that AM80 worsens inflammation in our NAFLD model. The HFD+AC261066 mice showed no macrophage positivity, similar to the chow-fed mice, confirming our previous findings [17]. There is evidence that AM80 may be a potential drug for diseases characterized by inflammation, such as rheumatoid arthritis and autoimmune uveoretinitis [68]^,^ [66], and type 1 diabetes [21]. In a mouse model of rheumatoid arthritis, which includes pulmonary complications, AM80 given at a dose of 3 mg/kg for 10 days inhibited the pro-inflammatory T follicular helper and Th17 cells and improved systemic inflammation [68]. Similarly, AM80, given at the same dose but for 21 days, induced Foxp3+ T regulatory cells and decreased IFNγ and IL17-producing Th17 in an experimental model of autoimmune uveoretinitis [66]. Another group investigated the role of AM80 in type 1 diabetes in mice [21] and found that one of the AM80 doses used in their study, 1 mg/Kg for 19 days, reduced hyperglycemia and insulitis, a typical type 1 diabetes consequence in which there is a profuse infiltration of T lymphocytes into the pancreas insulin-secreting β-cells, with progressive destruction of the insulin-producing cells. Morohoshi et al. [67] reported that in the same type 1 diabetes mouse model used by Miwako et al [21], used to study autoimmune thyroiditis in mice, AM80 did not show any anti-inflammatory effects. Thus, AM80 may display dose and tissue-specific effects which have not yet been delineated.

Given the increasing body of data demonstrating a role for retinoids in the pathophysiology and treatment of obesity-related disorders, the divergent metabolic effects of the RARα and RARβ2 specific agonists AM80 and AC261066 presented in this work reinforce the therapeutic potential of retinoids, but, equally important, also underscore the need for further preclinical studies to determine the therapeutic relevance and potential limitations of isotype-specific RAR agonists in the treatment of NAFLD and other obesity related disorders.

## Supporting information

S1 Fig The RARα agonist AM80 and RARβ2 agonist AC261066 do not affect serum triglycerides. Fasting serum triglycerides in chow, HFD, HFD+AM80, and HFD+AC261066 treated mice (4 mice per group). Mice were treated as in Fig 1. Serum triglycerides were measured as indicated in the Methods section. n.s. = not significant (p > 0.05).
(TIFF)
