# Supplementary figures and images for: Effects of AM80 compared to AC261066 in a high fat diet mouse model of liver disease
Source: PLoS One. 2019 Jan 24;14(1):e0211071. doi: 10.1371/journal.pone.0211071 (PMC6345457; doi:10.1371/journal.pone.0211071)

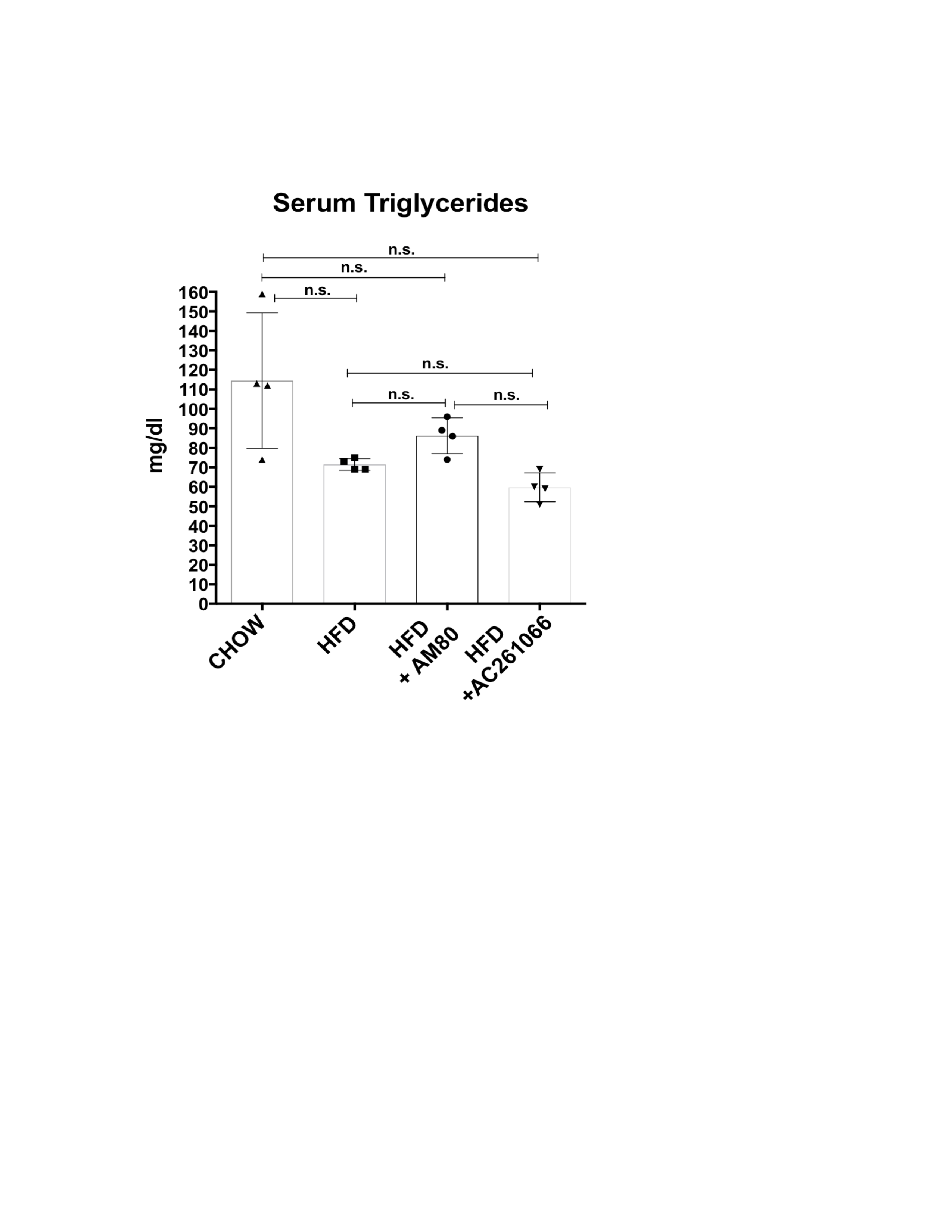

Supplement: S1 Fig — Fasting serum triglycerides in chow, HFD, HFD+AM80, and HFD+AC261066 treated mice (4 mice per group). Mice were treated as in Fig 1. Serum triglycerides were measured as indicated in the Methods section. n.s. = not significant (p > 0.05). (TIFF) [file pone.0211071.s001.tiff]
